# Human papillomavirus circulating tumor DNA: a diagnostic tool in squamous cell carcinoma of unknown primary—a pilot study

**DOI:** 10.3389/fonc.2024.1376595

**Published:** 2024-04-02

**Authors:** Amani Kais, Stell Patadji Santiago, Peng Cheng Han, David A. Clump, William A. Stokes, Tanya Fancy, Ruifeng Cui, Elizabeth Martin, Meghan T. Turner

**Affiliations:** ^1^ Department of Otolaryngology-Head and Neck Surgery, West Virginia University School of Medicine, Health Sciences Center, Morgantown, WV, United States; ^2^ Department of Pathology, West Virginia University School of Medicine, Health Sciences Center, Morgantown, WV, United States; ^3^ Department of Radiation Oncology, West Virginia University School of Medicine, Health Sciences Center, Morgantown, WV, United States

**Keywords:** squamous cell carcinoma of unknown primary, oropharyngeal cancer, diagnosis, HPV, ctDNA, biomarker, algorithm, transoral robotic surgery

## Abstract

**Introduction:**

Neck mass is the most common presentation of human papillomavirus-related (HPV-related) oropharyngeal squamous cell carcinoma (OPSCC). Recently, circulating tumor HPV-DNA (ctHPVDNA) assays have been developed to detect active OPSCC. This pilot study investigates the diagnostic accuracy of ctHPVDNA in establishing HPV status for known *vs.* unknown OPSCC presenting as a neck mass.

**Methods:**

A single-institution pilot study was conducted on all patients with OPSCC presenting as a neck mass between 2021 and 2022. The diagnostic accuracy of ctHPVDNA was compared to that of standard diagnostic procedures used to obtain HPV status according to the American Society of Clinical Oncology (ASCO) guideline for squamous cell carcinoma of unknown primary (SCCUP). Sensitivity, specificity, positive predictive value (PPV), and negative predictive value (NPV) of ctHPVDNA were calculated.

**Results:**

A total of 27 patients were included; 70.4% were current or former smokers, 48.1% (N = 13) had identifiable primaries, and 51.9% (N = 14) had SCCUP. Four patients with known primaries required operative direct laryngoscopy with biopsy (DLB) to establish HPV status. Two patients with SCCUP underwent diagnostic transoral robotic surgery (TORS) to establish HPV status and localize the primary. Twelve patients underwent therapeutic TORS and neck dissection. The gold standard for HPV status was based on final histopathologic p16 or HPV *in situ* hybridization (ISH) staining during workup/treatment. ctHPVDNA had 95.8% sensitivity, 100% specificity, 100% PPV, and 75% NPV in predicting HPV-positive OPSCC in the whole sample. Binary logistic regression model using ctHPVDNA results to predict HPV-positive OPSCC was significant (−2 log likelihood = 5.55, χ^2 ^= 8.70, p <.01, Nagelkerke’s R squared = .67). Among patients with identifiable primaries, all patients had HPV-positive tumors on final pathology, and ctHPVDNA was positive in 100%. In the unknown primary patients, ctHPVDNA had 90.9% sensitivity, 100% specificity, 100% PPV, and 75% NPV.

**Discussion:**

ctHPVDNA demonstrated good diagnostic accuracy for both known and unknown primaries. Incorporation of ctHPVDNA into the diagnostic algorithm for SCCUP may reduce the need for multiple procedures to establish HPV status.

## Introduction

The diagnostic approach and management of neck mass have changed drastically during the last century. Hayes Martin was the first to recognize that cervical metastasis was a common presentation of squamous cell carcinomas (SCCs) of the aerodigestive tract in 1944 (1). This realization led Martin to introduce a new approach to the workup of neck masses, advocating for physical examination with a headlamp and direct laryngoscopy with biopsy of the primary instead of excisional biopsy (2).

Much has changed since the time of Hayes Martin. Squamous cell carcinoma of unknown primary (SCCUP) is now rarely due to smoking and drinking-related laryngeal and hypopharyngeal cancers (3–5). Instead, human papillomavirus (HPV) oropharyngeal and Epstein–Barr virus (EBV) nasopharyngeal carcinomas are the most likely sources of SCCUP, which is reflected in the recently published American Society of Clinical Oncology (ASCO) guideline for diagnosis and management of SCCUP (6).

The current workup begins with a physical examination, fiberoptic flexible in-office nasopharyngoscopy, and fine-needle aspiration (FNA) biopsy followed by immunohistochemical (IHC) p16 nuclear staining and/or DNA/RNA *in situ* hybridization (ISH) for high-risk HPV DNA/RNA, and/or EBV-encoded small RNAs. FNA of the neck mass can both be diagnostic and narrow the location of the primary to the oropharynx or nasopharynx. However, FNA biopsy in the workup of HPV-positive oropharyngeal squamous cell carcinoma (OPSCC) has limitations. HPV-related cervical metastases are often cystic with significant necrosis, which can lead to indeterminate p16 staining patterns and a lack of nuclear material for HPV DNA/RNA ISH studies in as many as 49.5% of patients (7). For this reason, direct laryngoscopy with biopsy (DLB) of suspicious lesions is still needed but will only identify the primary only 20% of the time (8). Localization of the primary has been associated with improved overall survival (9). PET-CT can be of little use in the oropharynx, particularly for primaries smaller than 1 cm. Therefore, in cases where there remains no obvious tumor, transoral robotic surgery (TORS) is currently recommended to find microcarcinomas in the oropharynx and help target radiation therapy (6). Using this algorithm, 80% of SCCUPs should be found (9).

Recently, circulating tumor HPV DNA (ctHPVDNA) detectable in cell-free plasma has been developed as a biomarker for active HPV-positive cancers (10). Thus far, it has been used as a marker for treatment response and recurrence after chemoradiotherapy (11) and as a prognostic marker for recurrence after surgical treatment of OPSCC (12). Research examining the use of ctHPVDNA for diagnostic purposes has recently been conducted in non-smoking populations and has found a positive predictive value of 98.4% (13). To date, the diagnostic accuracy in SCCUP has not been specifically assessed. Given that delay in diagnosis is common for SCCUP, we suspect that ctHPVDNA may offer an improvement compared to current diagnostic methods. The present study aims a) to compare the diagnostic accuracy of ctHPVDNA in known *vs.* unknown primaries (5) and b) to describe how this test may fit into the diagnostic algorithm for SCCUP.

## Materials and methods

**Figure 1:**
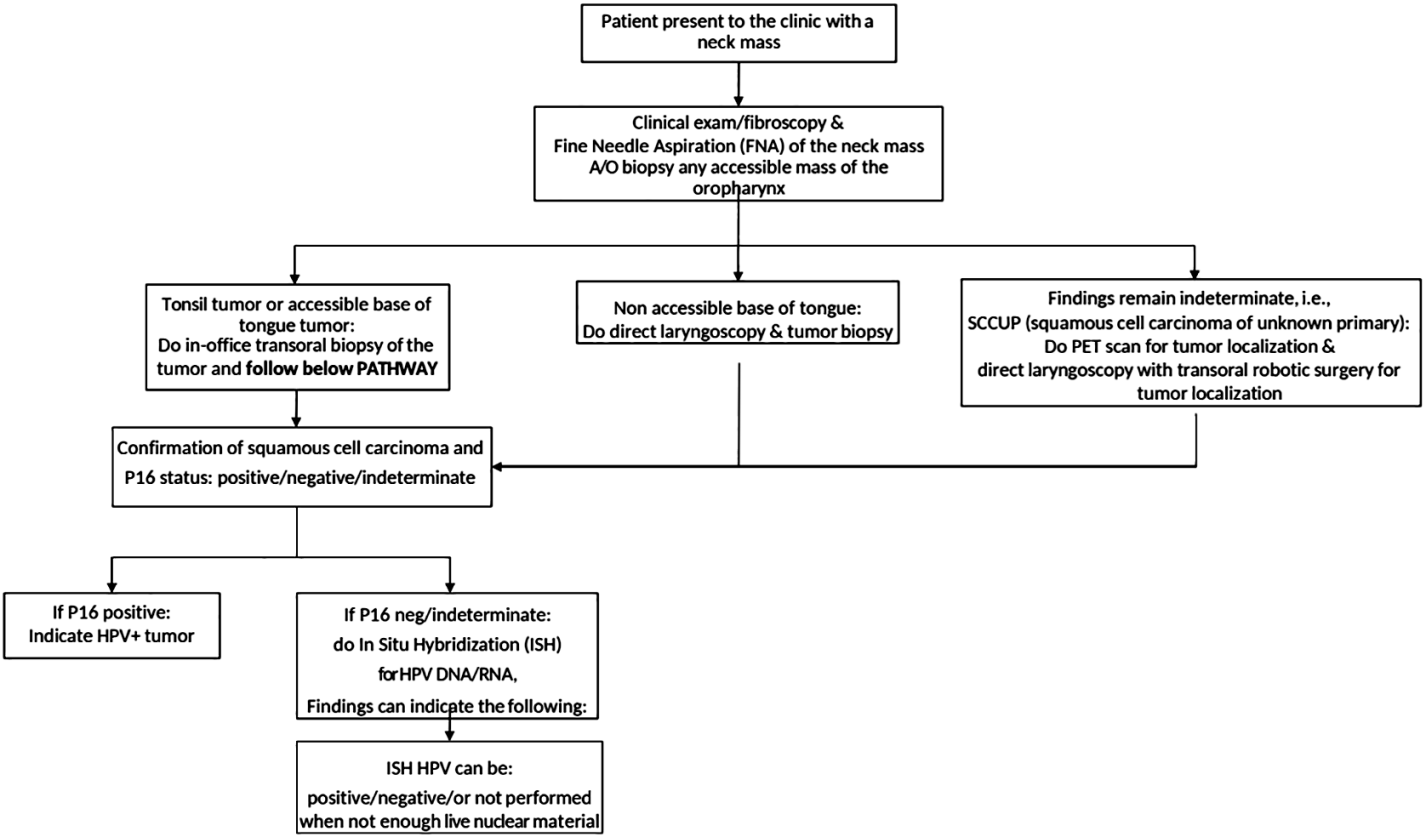
The flowchart describing the diagnostic workup of patients presenting to the otolaryngology clinic.

This pilot study is a retrospective review of prospectively collected data from a tertiary referral cancer center in West Virginia. Institutional Review Board approval was obtained for the purposes of the study (protocol: #2201496852). Due to the retrospective, observational nature of the study, the need for individual informed consent was waived. Twenty-seven consecutive patients with OPSCC presenting as a neck mass from January 2021 to October 2022 were included. All patients presenting for other reasons (incidentally found mass on imaging, hemoptysis, otalgia, dysphagia, and odynophagia) were excluded. All patients underwent routine diagnostic tests and procedures to obtain a diagnosis, determine HPV status, and stage the patient. The diagnostic workup flowchart is presented in **Figure 1**.

### Diagnostic procedure for identified primary

For visible tonsil primaries, in-office transoral tonsil biopsy and/or FNA of the neck mass was performed. For base of tongue (BOT tumors, in-office transoral biopsy was performed if tolerated as well as FNA. After a confirmatory diagnosis of SCC on pathology, p16 IHC was performed. If p16 staining was indeterminate or negative, cell blocks were sent for HPV-ISH (NeoGenomics Laboratories, Inc., Rochester, MN, USA). Patients underwent DLB of the primary tumor if HPV status remained unknown after office-based procedures. PET-CTs were performed in all patients for staging purposes.

### Diagnostic procedure for unidentified primary

**Figure 2:**
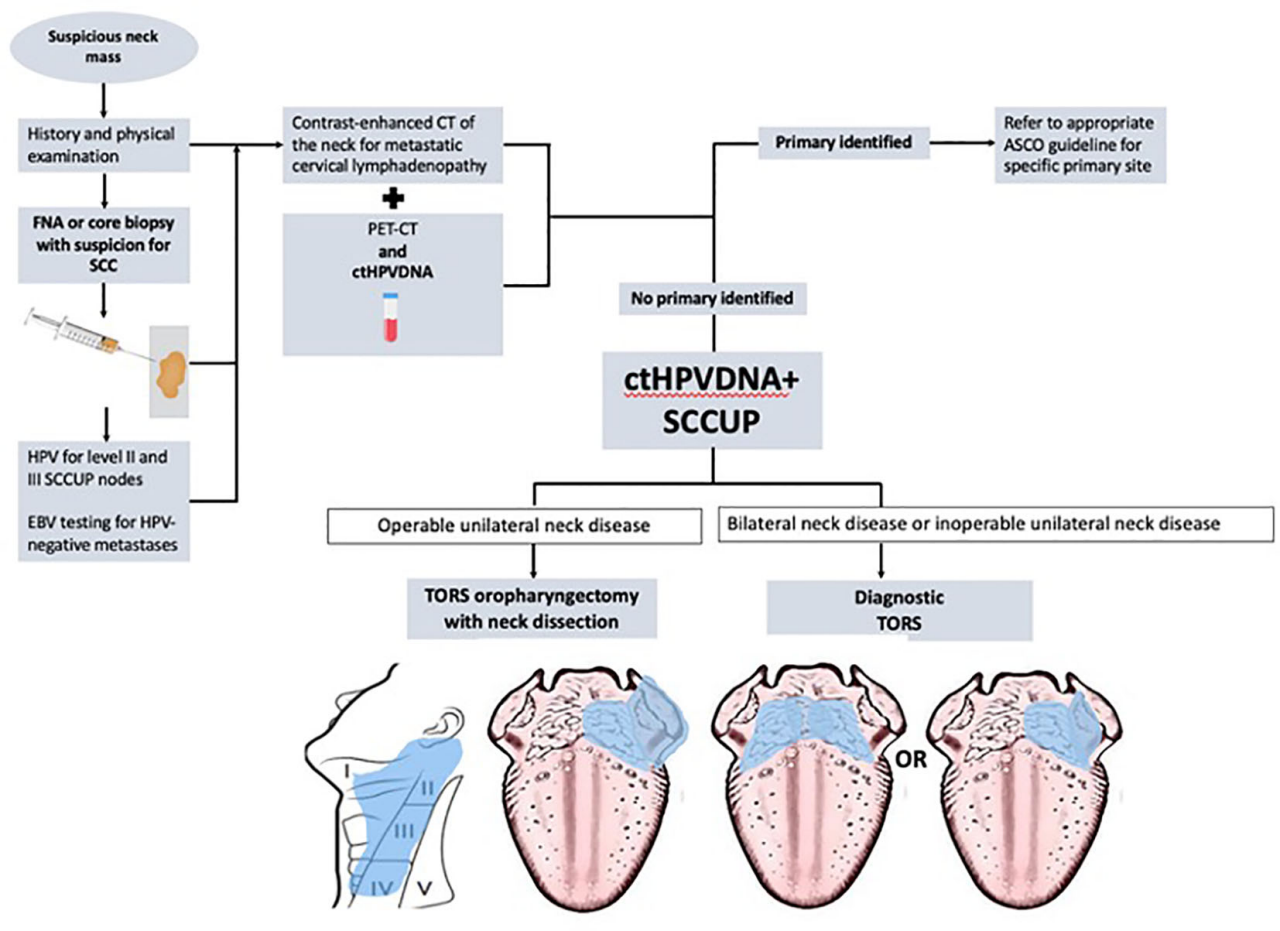
The flowchart describing the inclusion of the circulating tumor HPV-DNA (ctHPVDNA) test within the diagnostic algorithm of squamous cell carcinoma of unknown primary (SCCUP).

When no primary was identified on examination and endoscopy, FNA biopsy of the neck mass with in-office cytopathologic review was performed with hematoxylin and eosin staining. After cytopathologic diagnosis of SCC, p16 IHC was performed and considered positive if >70% of the nuclear material was positive. FNAs were considered indeterminate when p16 staining yielded <70% and exhibited nuclear positivity. HPV-ISH staining was performed when the p16 status was indeterminate or negative. PET-CTs were performed in all patients. All patients with SCCUP underwent TORS (diagnostic *vs.* definitive) to localize the primary and/or obtain HPV status. Patients with SCCUP were offered diagnostic TORS (for inoperable neck disease) or definitive TORS with neck dissection (for operable neck disease). Direct laryngoscopy was performed immediately prior to TORS for all unknown primaries, but random biopsies were not performed according to the recent ASCO guideline (6). Diagnostic TORS consisted of ipsilateral mucosectomy (tonsillectomy with hemi-tongue base mucosectomy) for unilateral neck disease or base of tongue mucosectomy for bilateral neck disease per the ASCO guideline. Definitive TORS consisted of ipsilateral pharyngectomy (radical tonsillectomy with the ipsilateral base of tongue mucosectomy) for unilateral lateral neck disease. See **Figure 2**.

### Diagnostic accuracy of ctHPVDNA

For all patients, ctHPVDNA liquid biopsy was performed (after a diagnosis of SCC was obtained from the neck or primary) using a NavDx^®^ testing kit (Naveris, Inc., Boston, MA, USA), which identifies amplicons of the E6/E7 proteins produced by high-risk HPV strains (16, 18, 31, 33, and 35) in cell-free plasma. Diagnostic accuracy was assessed by calculating the sensitivity, specificity, positive predictive value (PPV), and negative predictive value (NPV) of detectable ctHPVDNA compared to the gold standard (14, 15). The final pathologic HPV status (p16 and/or HPV-ISH status of the primary and/or neck mass) after complete workup was used as our gold standard.

### Treatment

After complete workup, patients with known primaries were offered surgery or standard definitive chemoradiation per guidelines. The percentage of patients undergoing TORS alone, TORS with adjuvant radiotherapy, and TORS with adjuvant chemoradiotherapy was calculated between known and unknown primary groups. Recommendations for adjuvant therapy followed recommendations based on pathologic risk stratification from the recent Eastern Cooperative Oncology Group 33-11 trial (15).

## Results

The sample consisted of 27 patients and was predominantly male (77.8%) with a mean age of 62.3 (SD = 10.3; range 37–79). Of these patients, 70.4% were current or former smokers with a >10 pack-year history. The remaining 29.6% had no smoking history. After routine physical examination and flexible laryngoscopy, 48.1% (N = 13) had an identifiable primary, while the remainder (N = 14) had SCCUP. The tumor characteristics and diagnostic procedures for the cohort are presented in **Table 1**.

**Table 1:** Tumor characteristics and diagnostic procedures.

	Number	Percentage
Site of primary		
Total Known Tonsil	27 13 8	100% 48.1% 29.60%
Base of tongue	5	18.50%
Unknown (SCCUP)	14	51.90%
Tumor size (in mm)		
Mean in known primary St Dev.	23.2 11.3	
Mean in unknown primary St Dev.	11.7 4.7	
Diagnostic tests/procedures used		
FNA for known primary (BOT) P16 IHC-positive HPV-ISH-positive Non-diagnostic	5 2 1 2	38.46% 40% 20% 40%
FNA for SCCUP Non-diagnostic p16 IHC-positive p16 IHC/HPV-ISH-negative	14 4 5 1	100% 10.5% 35.7% 7.1%
HPV-ISH-positive	4	28.6%
Diagnostic yield of FNA in entire cohort P16 status of primary tumor Biopsy known primary (p16 status)	13 17 13/13	68.4% 63.0% 100%
Tonsil (in-office biopsy) Base of tongue (in-office biopsy)	8/8 1/5	100% 20%
Base of tongue (operative DLB)	4/5	80%
TORS for SCCUP (p16 status) Contralateral tonsillectomy* PET scan in known primary	4/14 1/14 13	28.6% 7.1% 100%
PET scan in SCCUP	14	100%
Localizing	8	57.10%
Non-localizing	6	42.90%
TORS for SCCUP Diagnostic for SCCUP	2	14.30%
Therapeutic for SCCUP ctHPVDNA	12 27	85.7% 100%

*The patient who had a 45-mm visible tonsil mass had a non-typical presentation with bilateral neck disease and ended up having multiple synchronous primaries with small unknown primary in the contralateral tonsil.

SCCUP, squamous cell carcinoma of unknown primary; FNA, fine-needle aspiration; ISH, in situ hybridization; DLB, direct laryngoscopy with biopsy; TORS, transoral robotic surgery.

### Diagnostic procedures for known primaries

Thirteen had identifiable primary tumors after thorough physical examination and laryngoscopy. Eight patients (61.5%) had a visible tonsil lesion or mass and were able to undergo in-office transoral tonsil biopsy. Five patients (38.5%) had visible BOT masses, only one of which was accessible for in-office biopsy; four underwent operative DLB. Biopsy of the primary yielded a definitive p16 status 100% of the time (N = 13).

Five patients with base of tongue primaries also underwent FNA of the neck mass, and all FNAs yielded a diagnosis of SCC. However, only one was p16-positive, and four were indeterminate. Of the four indeterminate FNAs, two were HPV-ISH-positive, and two did not have enough live nuclear material for testing. There were no p16-negative or HPV-ISH-negative results. Overall, the diagnostic yield for FNA with ISH in a known primary was 60%. The diagnostic yield for performed testing is presented in **Table 2**.

**Table 2:** Diagnostic yield.

	Whole sample N = 27		Known primaries N = 13		Unknown primaries N = 14	
	#	%	#	%	#	%
FNA p16 results*	19		5		14	
Non-diagnostic	2	10.5%	0	0%	2	14.3%
Positive	6	31.6%	1	20.0%	5	41.7%
Indeterminate	10	52.6%	4	80.0%	6	50.0%
Negative	1**	5.3%	0	0%	1**	8.3%
HPV-ISH results*	11		4		7	
Positive	6	54.5%	2	50.0%	4	57.1%
Not enough viable material	4	36.4%	2	50.0%	2	28.6%
Negative	1**	9.1%	0	0%	1**	14.3%
Primary tumor p16 Results*	17		13		4	
Positive	15	90.5%	13	100%	2	50.0%
Negative	2	9.5%	0	0%	2	50.0%
Final HPV status	27		13		14	
Positive	24	88.9%	13	100%	11	78.6%
Negative	3	11.1%	0	0%	3	21.4%
ctHPVDNA results	27		13		14	
Positive	23	85.2%	13	100.0%	10	71.4%
Negative	4	14.8%	0	0%	4	28.6%
Sensitivity		95.8%				90.9%
Specificity		100%				100%
PPV		100%				100%
NPV		75.0%				75.0%
Final pathology (AJCC 8th ed.)						
T0	1	3.7%	0	0%	1	7.1%
T1	17	63.0%	5	38.5%	13	92.9%
T2	7	25.9%	6	46.1%	0	0%
T3	2	7.4%	2	15.4	0	0%
N1	24	88.	12	92.3%	12	85.7%
N2	2		1	7.7%	1	7.1%
N3	1		0	0%	1	7.1%

*Some patients had multiple tests.

**The p16-negative sample was also HPV-ISH-negative.

FNA, fine-needle aspiration; ISH, in situ hybridization; Final HPV status combines results from FNA p16, tumor p16, and HPV-ISH; PPV, positive predictive value; NPV, negative predictive value; HPV, human papillomavirus; ISH, in situ hybridization; ctHPVDNA, circulating tumor HPV-DNA; AJCC, American Joint Committee on Cancer.

### Diagnostic procedures for unknown primaries

Fourteen patients had no identifiable primary after the initial workup. All patients underwent an FNA biopsy of a cervical lymph node. Of those FNA biopsies, two were non-diagnostic. Twelve had FNA biopsies yielding a diagnosis of SCC, of which five were p16-positive, six were indeterminate, and one was p16-negative. HPV-ISH testing was performed on the seven patients with indeterminate or negative p16 staining. Of the indeterminate samples, four of those were HPV-ISH-positive, and two did not have enough live nuclear material for testing. The p16-negative sample was also HPV-negative on ISH. The overall diagnostic yield of FNA in SCCUP was 71.4%. All patients with SCCUP underwent PET-CT. Of these, 57.1% were localizing. TORS identified the primary in 13 patients (92.8%), all of whom were HPV-positive on final HPV status. The one patient whose primary was not found had a p16-negative, HPV-ISH-negative, and ctHPVDNA-negative neck mass. See **Table 2**.

### Treatment

After counseling on their treatment options, eight patients (29.6%) chose to proceed with definitive non-surgical therapy. Four of the known primary patients (all with base of tongue tumors) required operative DLB to determine HPV status. Two of the unknown primaries (14.39%) underwent diagnostic TORS for localization of the primary, after which both were localized. These two patients had large-volume neck disease (N2 and N3), and both underwent definitive non-surgical treatment.

Primary surgical treatment was chosen by the remaining 19 patients (70.4%): eight were known primaries, and 11 were unknown primaries. The mean tumor size for unknown primary tumors was 11.7 mm (SD = 4.7 mm). The mean tumor size for known primaries was 23.2 mm (SD = 11.3 mm). Among the 19 patients who underwent surgery, 10 patients (52.6%) underwent TORS alone, six patients (31.6%) had adjuvant radiotherapy, and three patients (15.8%) had adjuvant chemoradiotherapy. Among the 12 patients with SCCUP who chose to undergo definitive TORS and neck dissection, the primary was identified in 11 patients, and six (50%) were able to undergo surgery alone.

### Diagnostic utility of ctHPVDNA

ctHPVDNA liquid biopsy yielded results for all patients in the sample, i.e., 27 results. ctHPVDNA had a sensitivity of 95.8%, specificity of 100%, PPV of 100%, and NPV of 75% in predicting HPV-positive OPSCC diagnosis in the whole sample. The binary logistic regression model using test results from ctHPVDNA to predict HPV-positive OPSCC diagnosis from the gold standard was significant (−2 log likelihood = 5.55, χ^2 ^= 8.70, p <.01, Nagelkerke’s R squared = .67). Among the patients with an identifiable oropharyngeal primary, all patients had HPV-positive tumors on final pathology, and ctHPVDNA was positive in 100%. In the unknown primary patients, ctHPVDNA had 90.9% sensitivity, 100% specificity, 100% PPV, and 75% NPV.

## Discussion

This study is the first to compare the diagnostic accuracy of ctHPVDNA in known *vs.* SCCUP of the oropharynx. For patients with identifiable oropharyngeal primaries after physical examination and flexible laryngoscopy, 100% of patients with HPV-positive tumors had positive ctHPVDNA. Using the standard workup, 30.7% (4/13) of patients with known primaries required DLB solely in order to obtain HPV status. ctHPVDNA added to the diagnostic algorithm could reduce the number of operative biopsies for patients with identified primaries. For SCCUP, FNA biopsy with p16 staining and HPV-ISH demonstrated high rates of indeterminate findings in our patient sample, for a diagnostic yield of 71.4% in patients with SCCUP. For patients with unknown primary tumors, ctHPVDNA had 90.9% sensitivity, 100% specificity, 100% PPV, and 75% NPV. In this way, ctHPVDNA provided a more accurate diagnosis of HPV-related SCCUP than the standard workup.

Incorporating the use of ctHPVDNA into the ASCO diagnostic algorithm guidelines may help refine the application of TORS in SCCUP (**Figure 2**). First, used in this way, ctHPVDNA may help reduce the total number of procedures required to obtain HPV status in unknown primaries since multiple FNAs and sometimes operative biopsies are often required. Second, for patients with SCCUP and non-operable neck disease, the presence of ctHPVDNA may help encourage the use of diagnostic TORS when FNA biopsy is non-diagnostic or indeterminate per the ASCO guidelines in the hope of finding the primary, targeting radiation, and improving survival (8).

Finally, in select patients with unidentified primaries, ctHPVDNA use may reduce the number of diagnostic TORS procedures in favor of therapeutic TORS procedures. Diagnostic TORS comes with significant pain and a 5% risk of oropharyngeal hemorrhage (9). Therefore, reducing the total number of diagnostic TORS procedures may reduce this morbidity. In this study, only 14.3% (2/14 patients with SCCUP) of patients underwent purely diagnostic TORS procedures, favoring therapeutic TORS with neck dissection when HPV-positive disease was confirmed. Using ctHPVDNA to determine HPV status, SCCUP patients with a single metastasis who were treated with definitive TORS and neck dissection were able to avoid adjuvant therapy 50% of the time. Since there is often a delay in diagnosis for SCCUP, ctHPVDNA used to obtain HPV status could potentially reduce the time from diagnosis to completion of treatment for SCCUP.

### Limitations

The findings of this study should be interpreted in the context of several limitations. First, the sample size is small and may lead to overestimation of the accuracy of ctHPVDNA in known *vs.* unknown primaries since larger samples may be needed to capture rare diagnostic inaccuracies (false negatives or false positives) in SCCUP. However, a small sample size is a problem inherent to studies on SCCUP. Lastly, this is a single-institution experience in which there is a bias toward surgery and in which adjuvant therapy is adjusted based on the Eastern Cooperative Oncology Group (ECOG) 33-11 risk stratification. This may limit the generalizability of ctHPVDNA’s utility in practice at other institutions.

## Conclusion

This is the first study to investigate the diagnostic utility of ctHPVDNA in determining the HPV status in SCCUP. For the unidentified primary tumors, ctHPVDNA had 90.9% sensitivity, 100% specificity, 100% PPV, and 75% NPV. Incorporation of ctHPVDNA into the diagnostic algorithm for SCCUP may reduce the need for multiple procedures to establish HPV status and may facilitate definitive treatment.

## Data availability statement

The raw data supporting the conclusions of this article will be made available by the authors, without undue reservation.

## Ethics statement

The studies involving humans were approved by West Virginia University Institutional Review Board. The studies were conducted in accordance with the local legislation and institutional requirements. The ethics committee/institutional review board waived the requirement of written informed consent for participation from the participants or the participants’ legal guardians/next of kin because this was a retrospective chart review performed without contact with patients and involving no risk to patients.

## Author contributions

AK: Writing – original draft, Data curation. SS: Data curation, Formal Analysis, Writing – review & editing. PH: Data curation, Writing – review & editing, Formal Analysis. DC: Writing – review & editing. WS: Writing – review & editing. TF: Writing – review & editing. RC: Formal Analysis, Validation, Writing – original draft. EM: Formal Analysis, Writing – original draft. MT: Conceptualization, Formal Analysis, Methodology, Supervision, Writing – original draft, Writing – review & editing.
